# Paraplegia Following Decompression Sickness (Caisson Disease): A Journey to Near-Complete Recovery

**DOI:** 10.7759/cureus.117461

**Published:** 2026-10-05

**Authors:** Lisha Michel M, Kiran Kumar, Sharmila Banu Eashak Ali, Rozhina Ghanbari, Rifa Khan, Khadeeja Mahjoor

**Affiliations:** 1 Internal Medicine, Thumbay University Hospital, Ajman, ARE; 2 Physiotherapy, Thumbay Group (United Arab Emirates), Ajman, ARE; 3 General Practice, Thumbay Group (United Arab Emirates), Dubai, ARE; 4 Medicine and Surgery, Gulf Medical University, Ajman, ARE; 5 General Practice, Thumbay University Hospital, Ajman, ARE

**Keywords:** acute paraplegia, caisson disease, decompression sickness, neurological recovery, spinal cord injury

## Abstract

Decompression sickness (DCS), also known as “Caisson disease,” is a potentially life-threatening condition caused by the formation of inert gas bubbles during rapid decompression, most commonly affecting deep-sea divers. Neurological involvement, particularly spinal cord injury leading to paraplegia, represents one of the most severe manifestations. We report the case of a 44-year-old Filipino male professional diver who developed acute paraplegia following a routine dive. Despite adherence to decompression protocols, he experienced sudden-onset bilateral lower limb weakness during ascent. Initial management included oxygen therapy followed by hyperbaric oxygen therapy, after which he was referred for intensive multidisciplinary rehabilitation within a few days. At presentation, the patient exhibited significant motor weakness with Medical Research Council grading of 2/5 in the left lower limb and 3/5 in the right lower limb, sensory loss below the T8 level, impaired bladder and bowel control, and complete dependence for mobility. Over a period of approximately three months of structured physiotherapy, including conventional therapy, robotic-assisted rehabilitation, neuromuscular stimulation, and gait training, he demonstrated remarkable functional recovery. Muscle strength improved to 4/5, with restoration of independent ambulation, improved sensory function, and regained bladder control. This case highlights the importance of early recognition, prompt hyperbaric therapy, and intensive rehabilitation in achieving near-complete recovery following severe neurological DCS.

## Introduction

Decompression sickness (DCS) is a pressure-related disorder that occurs following a reduction in ambient pressure, resulting in the formation of inert gas bubbles within tissues and the circulation. These bubbles may produce mechanical vascular obstruction, endothelial injury, inflammation, and secondary tissue ischemia, with the musculoskeletal and nervous systems being particularly vulnerable [1,2].

Neurological decompression sickness (NDCS) represents a potentially severe manifestation and may involve the spinal cord, producing lower-limb weakness or paraplegia, sensory disturbances, and autonomic dysfunction, including bladder and bowel impairment [2-4]. Spinal cord involvement may occur through multiple mechanisms, including intravascular gas embolization, vascular compromise, and secondary ischemic injury. Although adherence to established decompression procedures reduces risk, DCS can occur despite apparently appropriate diving practices, reflecting the influence of individual and environmental factors [1,2].

Prompt recognition and administration of oxygen are fundamental components of the initial management of DCS, while hyperbaric oxygen therapy (HBOT) remains the principal definitive treatment for significant neurological manifestations [2,5-7]. The timing and clinical response to HBOT are important considerations, although neurological recovery may also reflect the natural course of the condition and other aspects of supportive care. Following acute stabilization, structured multidisciplinary rehabilitation may address motor weakness, impaired balance, gait dysfunction, and activities of daily living, but the independent contribution of specific rehabilitation modalities to neurological recovery in NDCS remains incompletely defined [3,4,8].

We report a case of severe spinal cord-predominant NDCS presenting with acute paraplegia, a T8 sensory level, autonomic dysfunction, and marked functional dependence, followed by substantial neurological and functional improvement after acute oxygen/HBOT treatment and an intensive multidisciplinary rehabilitation program. This case highlights the clinical presentation, multidisciplinary management, and documented functional recovery of severe NDCS while recognizing that the contribution of individual interventions cannot be established from a single case.

## Case presentation

A 44-year-old Filipino male, a professional deep-sea diver, performed a dive to a depth of approximately 30 meters for about 12 minutes. He initiated ascent according to standard decompression protocols; however, during the final phase of ascent at a shallow depth, he developed sudden numbness in both lower limbs followed by progressive weakness. There was no history of loss of consciousness or respiratory distress, although he reported mild disorientation. Immediate onboard oxygen therapy was administered, following which he was transferred to a nearby medical facility where HBOT was initiated, which remains the cornerstone of treatment for DCS. Within a few days, he was referred to a tertiary care center for further management and comprehensive rehabilitation.

Clinical findings

At the time of admission to the rehabilitation facility, the patient was conscious, well-oriented, and able to follow commands. Neurological examination revealed significant motor weakness in both lower limbs, with muscle strength graded as 2/5 on the left side and 3/5 on the right side. Deep tendon reflexes were exaggerated, and plantar responses were extensor, with clonus noted in the left lower limb. Sensory examination demonstrated reduced sensation below the T8 spinal level, consistent with spinal cord involvement reported in NDCS. The patient also exhibited autonomic dysfunction in the form of impaired bladder and bowel control, requiring catheterization. Trunk control was poor, and he was unable to maintain independent sitting or standing balance. Functionally, he was wheelchair-dependent and required maximal assistance for activities of daily living.

Thoracic spine MRI

MRI of the thoracic spine was performed during the acute evaluation but was limited by motion artefacts. The vertebral bodies demonstrated preserved height and marrow signal intensity. No significant posterior disc bulge or herniation and no spinal canal stenosis were identified. No compressive structural abnormality was demonstrated. In the context of the acute neurological presentation following the decompression event, these findings supported a non-compressive spinal cord process consistent with NDCS. The limited examination, however, may have reduced sensitivity for subtle intrinsic spinal cord abnormalities.

Management and rehabilitation

The patient initially received oxygen therapy followed by HBOT, which was continued alongside the rehabilitation program, as recommended in the management of DCS.

The patient underwent an intensive, structured rehabilitation program for approximately three months, with two rehabilitation sessions per day. Therapy was progressively advanced according to neurological recovery and functional tolerance. Initial therapy focused on lower-limb stretching and strengthening, trunk control, sitting balance, and prevention of secondary complications, followed by progressive weight-bearing, standing, balance and coordination training, and gait rehabilitation. Robotic-assisted therapy, antigravity treadmill gait training, neuromuscular electrical stimulation, sensory re-education, and hydrotherapy were incorporated progressively as functional capacity improved. The program ultimately progressed from assisted mobility to independent standing and ambulation.

At the three-month assessment, lower-limb motor strength had improved from 2/5 on the left and 3/5 on the right to 4/5 bilaterally. The patient progressed from wheelchair dependence with inability to stand or walk to independent standing and ambulation with good balance and coordination. Clonus was reduced, sensory function improved with only mild residual gluteal paresthesia, and bladder function recovered sufficiently to allow removal of the Foley catheter and independent self-voiding. Trunk stability and sitting and standing balance also showed marked improvement.

Functionally, the patient progressed from requiring maximal assistance for activities of daily living to achieving independence (Table 1).

**Table 1:** Clinical status of the patient before and after undergoing extensive rehabilation.

Parameter	Initial status	Post-rehabilitation outcome (3 months)
Motor strength (lower limb)	Lower extremity: 2/5, right lower extremity: 3/5	Improved to 4/5 in both lower limbs
Mobility status	Wheelchair-dependent, unable to stand or walk	Independent standing and walking with good balance and coordination
Reflexes	Exaggerated plantar reflex (Babinski positive), clonus present	Clonus reduced, reflexes better controlled
Sensory examination	Reduced sensation below the T8 level	Improved sensation; mild residual paraesthesia (gluteal region)
Autonomic function	Reduced control; Foley catheter in situ	Foley removed; regained bladder control with self-voiding
Trunk control	Poor trunk stability	Markedly improved trunk control
Balance and coordination	Poor sitting, standing, and dynamic balance	Good balance and coordination
Functional status	Required maximal assistance for daily activities	Independent in activities of daily living

Follow-up and future plan

The patient was advised to continue regular physiotherapy on a daily basis with emphasis on dynamic control exercises and hydrotherapy to further enhance functional performance. Follow-up over the subsequent weeks was planned to monitor progress and adjust the rehabilitation program as needed. With continued improvement, the patient was gradually transitioned to higher-level performance training.

## Discussion

NDCS remains a rare but serious complication of diving and may result in long-term disability if not treated promptly [5]. Spinal cord involvement is particularly associated with significant morbidity due to the susceptibility of neural tissue to ischemic injury caused by intravascular and extravascular gas bubbles [6]. This case highlights the critical importance of early HBOT in reducing bubble size, improving oxygen delivery, and limiting secondary neurological damage. In addition, it emphasizes the essential role of early, intensive, and multidisciplinary rehabilitation in optimizing functional recovery. Recovery in such cases is influenced by several factors, including the severity of the initial neurological deficit, the timeliness of intervention, and the intensity and consistency of rehabilitation. The near-complete recovery observed in this patient underscores the potential for favorable outcomes even in severe neurological presentations when appropriate management is instituted.

The substantial neurological and functional recovery observed in this patient occurred following early medical management with oxygen therapy and HBOT, followed by an intensive multidisciplinary rehabilitation program. However, the contribution of each component cannot be independently determined from this single case. Recovery from NDCS may reflect the combined effects of acute treatment, the natural course of neurological recovery, and rehabilitation. Although the temporal association between treatment and functional improvement is clinically notable, this case does not establish a causal relationship between HBOT or any individual rehabilitation modality and neurological recovery. Further studies using serial standardized neurological and functional outcome measures are required to better characterize the contribution of these interventions.

## Conclusions

Severe spinal cord-predominant NDCS can result in substantial motor, sensory, autonomic, and functional impairment. In this case, the patient demonstrated marked neurological and functional recovery following acute oxygen and hyperbaric management, followed by an intensive multidisciplinary rehabilitation program. However, the relative contributions of HBOT, rehabilitation, and the natural course of NDCS cannot be determined from a single case. This case highlights the importance of prompt recognition, appropriate acute management, and structured multidisciplinary rehabilitation in the overall care of patients with severe NDCS, while recognizing the need for further studies using serial standardized neurological and functional assessments to better characterize recovery and treatment outcomes.
